# Time Reduction for SLM OFDM PAPR Based on Adaptive Genetic Algorithm in 5G IoT Networks

**DOI:** 10.3390/s23239310

**Published:** 2023-11-21

**Authors:** Esam A. A. Hagras, Sameh F. Desouky, Saad Aldosary, Haitham Khaled, Tarek M. Hassan

**Affiliations:** 1 Faculty of Engineering, Delta University for Science and Technology, Gamasa 35712, Egypt; esam.hagras@deltauniv.edu.eg; 2 Independent Researcher, Ottawa, ON K1S 5B6, Canada; samehfarahat@gmail.com; 3 Department of Computer Science, Community College, King Saud University, Riyadh 11437, Saudi Arabia; saldosary@ksu.edu.sa; 4 Department of Electronics and Communications, School of Engineering, Edith Cowan University, Perth, WA 6027, Australia; h.khaled@ecu.edu.au

**Keywords:** adaptive genetic algorithm, selected mapping, peak-to-average power ratio

## Abstract

In this paper, a new peak average power and time reduction (PAPTR) based on the adaptive genetic algorithm (AGA) strategy is used in order to improve both the time reduction and PAPR value reduction for the SLM OFDM and the conventional genetic algorithm (GA) SLM-OFDM. The simulation results demonstrate that the recommended AGA technique reduces PAPR by about 3.87 dB in comparison to SLM-OFDM. Comparing the suggested AGA SLM-OFDM to the traditional GA SLM-OFDM using the same settings, a significant learning time reduction of roughly 95.56% is achieved. The PAPR of the proposed AGA SLM-OFDM is enhanced by around 3.87 dB in comparison to traditional OFDM. Also, the PAPR of the proposed AGA SLM-OFDM is roughly 0.12 dB worse than that of the conventional GA SLM-OFDM.

## 1. Introduction

The 5G-enabled internet of things (5G-IoT) has received a lot of interest recently due to its affordability, efficiency, and coverage. The modulation technique plays an important role in the 5G-IoT performance. Because of its simple implementation and capacity to deal with multipath channels, the conventional modulation technology known as OFDM has been widely applied to a variety of communication protocols [1,2,3]. Despite being a multicarrier modulation, OFDM has the drawback of having a high peak-to-average power ratio (PAPR), which lowers the efficiency of the power amplifier and negates the benefits of its low power consumption and broad coverage [4]. For 5G-IoT systems, OFDM, therefore, might not be the ideal choice [5].

The PAPR reduction in OFDM systems has been addressed using a number of different strategies up to this point. There are two basic groups into which these techniques fall. One category calls for the side information (SI) to be transmitted in advance, which uses up valuable time–frequency resources in control channels in general. For instance, the partial transmit sequence (PTS) was suggested in [6], and the selective mapping (SLM) method was taken into consideration in [7]. High complexity is another significant downside for the chosen SLM and PTS approaches as a result of them being the best choice among many phase sequences. SI-free approaches fall under another category of PAPR reduction techniques [8,9,10,11,12]. In [8], the PAPR was decreased by physically clipping the transmitted signal, albeit at the expense of the bit error rate (BER) and out-of-band radiation performance degradation.

A peak-cancelling signal was used in [9] to generate a series of reserved tones, and the spectral efficiency was decreased as a result of the tonal reservation method’s use of them to lower the PAPR. Using extension matrices made up of amplitude extensions and phase rotations, an extended SLM for the PAPR reduction without SI was proposed in [10]. The construction of coset codes from a linear polar code was used in [11] to offer a polar coding-based SI-free SLM method. In addition, a blind adaptive trial receiver was shown for signal identification. In [12], the authors introduced a method of blind interleaving with signal space diversity that greatly lowers the PAPR in OFDM without the use of SI. In the recent past, a new embedded SLM-PTS combination approach for PAPR reduction of OFDM systems has been introduced [13]. According to [13], an effective embedded SLM-PTS combination scheme (ESP) is one that splits the OFDM signal into smaller blocks, performs SLM steps in each sub-block, and then moves on to the next step with the results of each sub-block. In [14], the authors proposed that the SLM, based on the chaotic biogeography-based optimization (CBBO) algorithm, give a practical solution for the problem of high PAPR that appears in generalized frequency-division multiplexing waveforms. In [14], according to experimental results, the CBBO-SLM scheme greatly lowers PAPR when compared to conventional SLM methods like conventional GFDM and OFDM-SLM. In [15], in order to minimize the PAPR value, the authors use the SLM with several matrices, including the Greatest Common Divisor (GCD), Random, Riemann, Lehmer, and Hanowa. According to simulation results, the SLM–Hanowa matrix performs better, reducing PAPR by 7.86 dB to 9.82 dB. A unique Divergence Selective Mapping (DSLM) and Divergence Partial Transmission Sequence (D-PTS) for 5G waveforms are presented in [16]. It can be shown in [16] that the suggested D-SLM and PTS reduce PAPR with minimal computing effort.

The genetic algorithm (GA) is widely used as an effective optimization technique to overcome the difficulty and complexity of finding the suitable phase-rotation factors of PAPR [17,18,19]. Also, it has been used in different applications, such as wall-following [20] and differential games [21]. Unlike many traditional optimization techniques, GAs do not require the computation of local derivatives to guide the search process; all they require is an objective function [22]. So, AGA is used in order to increase the convergence rate because the typical GA’s convergence rate is slow. In the mutation operator, the AGA is used with the assistance of Cauchy mutation. The Cauchy mutation operator was added to the GA to both improve GA performance and hasten the GA process. A meta-heuristic method called the AGA reduces the rate of natural evolution. It is most likely utilized to give rise to explanations for optimization and search issues. Using methods inspired by natural evolution, such as inheritance, mutation, selection, and crossover, it gives rise to explanations for optimization problems.

AGA is used in many applications, such as code design [23]. In [23], two operators are used, schemata crossover (SC) and allele gene adaptive mutation (AGAM) which are used in the code design. The AGA is used in the code design to enhance the performance of the traditional genetic algorithm (GA). To maintain the right level of diversity throughout the search process, the suggested AGAM specifically makes use of both global and local information about the population. To improve conventional crossover functioning, the SC makes use of high-performance schemata. Both the goal of maintaining population variety and the goal of simultaneously sustaining the GA’s convergence potential are achieved by combining the AGAM and SC. As a result, the SC- and AGAM-based genetic algorithm reduces the premature convergence that the GA is prone to, thus raising the likelihood of discovering the overall best solution.

The AGA is used in sensor applications. In [24], the AGA is used for adjusting sensor acquisition frequency. In order to decrease the power consumption of portable meteorological stations, the author in [24] suggests a method of adaptive modification for the data-acquisition approach for environmental monitoring. This method is based on the AGA. An acceptable weather curve can be fitted using data with a relatively low acquisition frequency by optimizing the daily acquisition approach. The experimental findings in a real-world setting demonstrate the algorithm’s capability to successfully optimize the acquisition approach. Also, the AGA is used in cryptosystems to improve random number selection. In [25], an optimal AGA-based hybrid signcryption algorithm for information security was proposed. The digital signcryption approach in [25] encrypts the original message using an elliptic-curve-cryptography algorithm that is based on the AGA. In this case, they introduced three games for security reasons and showed that attackers are unable to identify the security features of the signcryption technique. In addition, to ensure secure data transfer, the signcryption technique is examined with regard to brute force, man in the middle, and Denial-of-Service attacks (DoS).

In this paper, the AGA is applied to SLM-OFDM to find the best phase-rotation factors, resulting in a greater improvement in PAPR reduction when compared to OFDM and SLM-OFDM. In addition, AGA is used to solve the long selection time of the GA. Using GA alone, it takes a long time to select the phase-rotation parameters (off-line selection). The proposed AGA SLM-OFDM technique is used as an on-line selection of phase-rotation parameters. This paper is organized as follows: In Section 2, the SLM OFDM system is introduced. Section 3 describes the proposed AGA SLM-OFDM technique. Computer simulation and results are presented in Section 4. Finally, conclusions are drawn in Section 5.

## 2. SLM-OFDM System

### 2.1. OFDM System and PAPR Problem

A QPSK modulator is used to construct an OFDM symbol, which is made up of subcarriers modulated using constellation mapping. The serial-to-parallel (S/P) conversion is then used to convert these symbols into a parallel signal sequence of length *N* called $X_{k}$, *k* = [−(*N*/2), −(*N*/2) + 1, …, (*N*/2) − 1]. The transmitted OFDM symbol is then created using the IFFT and has *N* orthogonal subcarriers of equal bandwidth and Δf=1/T frequency spacing (*T* is the time duration of the OFDM symbol). The representation of the transmitted OFDM symbol is as follows [12]:

(1) $$x_{n}=\frac{1}{\sqrt{N}}\sum_{k=-\frac{N}{2}}^{\frac{N}{2}-1}X_{k}e^{\frac{j2\pi nk}{LN}}$$

where *L* is the oversampling factor (an integer 1); based on Equation (1), the sent signal PAPR is defined as follows [12]:

(2) $$PAPR=10\log_{10}\frac{\underset{0\leq n\leq LN-1}{max}[{\left|x_{n}\right|}^{2}]}{E[{\left|x_{n}\right|}^{2}]},$$

where *E* [·] denotes an operation with anticipated value.

### 2.2. SLM Technique

The basic idea behind the random search SLM-OFDM is to generate a set of suitable different OFDM symbols *X*^(*m*)^, 0 ≤ *m* ≤ *M* − 1, each of length *N*, with the *M* different OFDM symbols *X*^(*m*)^ representing the same data as the original OFDM symbol $x_{n}$, and then to send the signal with the lowest PAPR out of the collection. To create the set of OFDM symbols, the initial data block $X_{k}$, *k* = [−(*N*/2), −(*N*/2) + 1, …, (*N*/2) − 1] can be multiplied, element by element, with *M* different phase sequences (*m*), each of length *N*. The phase sequences *ψ*^(*m*)^ in question are indicated using [20]

(3) $$\psi^{\left(m\right)}=[\psi_{0}^{\left(m\right)},\psi_{1}^{\left(m\right)},\ldots ,\psi_{N-1}^{\left(m\right)}],0\leq m\leq M-1$$

The element-by-element multiplication of *X_k_* and *ψ*^(*m*)^, *0* ≤ *m* ≤ *M* − 1 is the modified OFDM symbol *x*^(*m*)^, which is represented by the IFFT as [20]

(4) $$x^{\left(m\right)}=IFFT\left\{X_{k}.\psi^{\left(m\right)}\right\}=\left[x_{0}^{\left(m\right)},x_{1}^{\left(m\right)},\ldots ,x_{N-1}^{\left(m\right)}\right].$$

A phase-rotation factor’s ability to reduce PAPR in an SLM system depends on how many there are (*M*) and how they are made [20]:

(5) $$X=\sum_{m=1}^{M}X_{m},m=1,2,\ldots ,M.$$

## 3. Proposed AGA SLM-OFDM Technique

**Figure 1:**
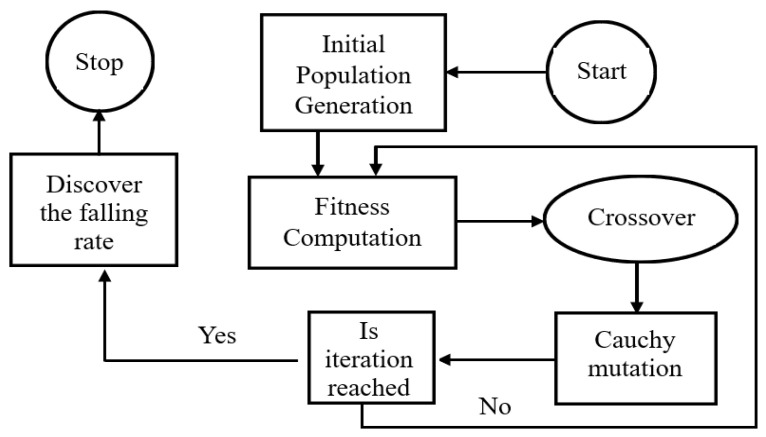
Flowchart of the AGA [25].

The AGA [25], an algorithm with meta-heuristics, reduces the natural evolution rate. It most likely produced explanations for optimization and search issues in the past. It gives birth to explanations for optimization issues utilizing methods inspired by natural evolution, such as the concepts of heredity, mutation, selection, and crossover. Figure 1 shows the flowchart of the AGA scheme.

Initial phase: The initial generation of the populations of the chromosomes $x_{i},\left(i=1,2,\ldots T\right)$ is random, where “T” denotes the population size. Certain integer values that were created at random and are less than the selected prime number $g_{i}$ can be found on the chromosome $x_{i}$.

Fitness function: We determine the fitness value of each parameter and then select the chromosome with the highest fitness value, where $f\left(i,j\right)=min(er(i,j))$, and er(i, j) are the rate of error of the t-th parameter.

Selection of chromosomes: The best T/2 chromosomes with the least fitness are used to select one or more parent chromosomes, and a new generation is produced.

Crossover: At the crossover rate of ($C_{r}$), which produces T/2 offspring, a single data point is crossed over. Every crossover operation involves the exchange of ${TC}_{r}$ genes between related parents.

Mutation: Individuals are perturbed probabilistically in order to effect change. When using the mutation operator, there may be a chance for some new features to appear as a result of chromosome changes. Cauchy mutation is used to mutate individuals using the equation below. Mutation is carried out based on a predetermined mutating probability. If we use Cauchy mutation, the random variable *x* will have a Cauchy distribution whose function is given as follows [25]:

(6) $$F\left(X\right)=\frac{1}{2}+\frac{1}{\pi }arctan(x)$$

Updation: The first chromosome is being replaced in the sixth stage with a new one. Following the mutation process, the original population’s chromosomes are replaced with the T/2 elected and new T/2 offspring chromosomes.

Termination criteria: Repeating the procedure makes sure that the termination requirements are satisfied.

**Figure 2:**
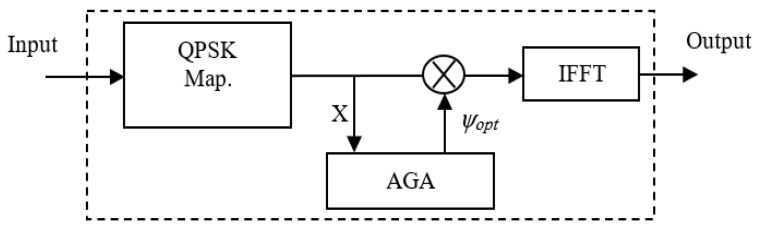
The proposed AGA SLM OFDM technique.

**Figure 3:**
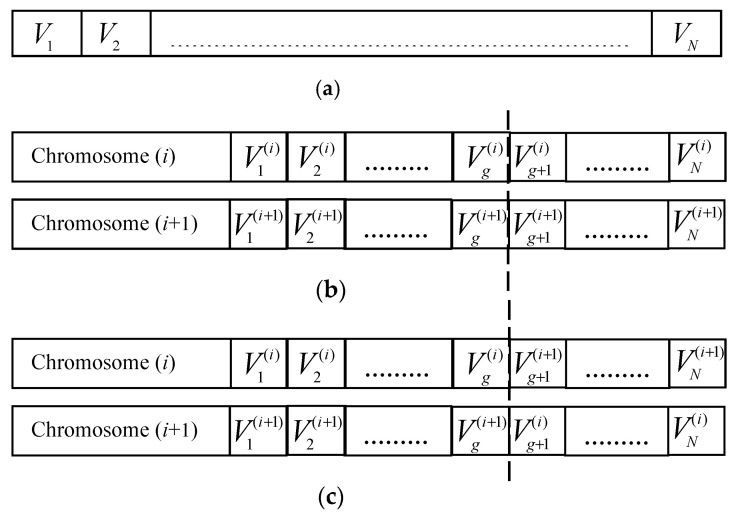
Crossover operation: (**a**) A phase-rotation vector coded into a chromosome, (**b**) an old chromosome, and (**c**) a new chromosome.

This section investigates how AGA can be used for phase optimization of SLM-OFDM system. In [11,12,13,14,15,16,17], GAs have been used for off-line tuning of the phase-rotation factors of PAPR. Although the used method finds suitable values, it takes a comparatively long time to do that. In addition, the system suffers from complexity. The proposed AGA SLM-OFDM technique, shown in Figure 2, is used for on-line tuning of the phase-rotation factors to minimize the complexity of the system; the PAPR of transmitted signals; and then the time taken in phase selection. In the proposed AGA SLM-OFDM technique, the phase-rotation vector has a length of *N* carriers. Therefore, a phase rotation will be coded into a chromosome with a length of *N* = 128 genes, as shown in Figure 3a. In the coding process, we use real numbers {1, −1, *j*, −*j*}. The P chromosomes make up the population (coded phase-rotation vectors). Using crossover and mutation processes, we create new chromosomes throughout the reproduction process. As shown in Figure 3b,c, a cross replacement is carried out after a random gene, g, is selected between 1 and (*N* − 1), and a set of parents are chosen for the crossover operation.

In the mutation process, in order to prevent the fitness function from reaching a local minimum, a chromosome is constructed at random. With the help of the fitness function, we can now test a new population. Up until a termination requirement is satisfied, the genetic process is repeated. A number of conditions can cause the genetic process to halt, including [18] that the maximum number of iterations has been reached; a fitness threshold has been reached; the maximum time has been reached; and a combination of the aforementioned conditions. In our work, the procedure is repeated when the allotted number of iterations has been reached. It is necessary to resolve the following optimization problem in order to reach the optimal phase factors that lower the AGA SLM-OFDM PAPR value using the suggested AGA SLM-OFDM method [18]:

(7) $$\psi_{opt}=\arg\underset{p}{min}\frac{\underset{0\leq n\leq N-1}{max}[{\left|x_{n}\right|}^{2}]}{E[{\left|x_{n}\right|}^{2}]},$$

where *ψ_opt_* is the optimum phase-rotation factors [18]:

(8) $$x_{k}=IFFT\{X_{k}.\psi_{opt}\}$$

Algorithm 1 describes the proposed AGA SLM-OFDM learning method.
**Algorithm 1**: AGA SLM-OFDM technique**1**.Initialize a set of chromosomes (randomly) in a population, *P*. **2**.***For*** each iteration *i* of *I*.
(*a*)***For*** each chromosome *p* of P.
(i)Construct a rotation phase from the *p*th chromosome.(ii)Evaluate the PAPR.(iii)Calculate the *p*th fitness value using (6).(*b*)***End***(*c*)Sort the chromosomes based on their fitness values.(*d*)Choose a subset of the sorted chromosomes for the next generation.(*e*)Using crossover and mutation operations, create the remainder of the new generation.
**3**.***End***

## 4. Simulation Results

The PAPR performance for OFDM, SLM OFDM, the traditional GA-SLM-OFDM, and the suggested AGA-SLM-OFDM approaches are examined using computer simulations. In the simulation study, 10^4^ random OFDM signals are produced in the simulations to find the CCDF. The values of the phase-rotation-factor elements (*m*) are (1, *j*, −1, −*j*). We employ a core i5 CPU running at 3.2 GHz and 4 Gigabytes of Memory. Table 1 lists the simulation parameters that were employed throughout the comparison analysis.

The complementary cumulative distribution function value determines the capability to reduce PAPR (CCDF). The probability that the PAPR of an OFDM symbol will be above the defined threshold PAPR_o_ is known as the CCDF and is represented as follows [18]:

(9) $$CCDF[PAPR(x_{n})]=prob(PAPR(x_{n})>PAPR_{o})$$

Several parameters for the AGA and GAs must be predetermined. As a result, we examined several parameter values and parameter value combinations using computer simulation before selecting the ones that produced the best results. We combine several iteration numbers and population sizes to determine the number of iterations and the population size. The AGA and GA simulations are then run 100 times, and the fitness levels for each combination throughout the 100 runs are averaged. We sort the average of the fitness values, and then we choose the combination at which the improvement in the average fitness value is less than 2%. The iteration numbers that we tested were started from 2, to 10. The population sizes that we tested were started from 10, to 100. The comparison between the AGA-SLM and GA-SLM with selected different numbers of iteration and different population sizes is shown in Table 2, Table 3 and Table 4. The time (second) and PAPR (dB) saving between the proposed AGA SLM OFDM and the GA SLM OFDM are calculated at the same number of iteration and the same number of population size.

In Table 2, the PAPR for the GA SLM OFDM system began at 7.77 dB at a population size of 10, and it reached a maximum of 5.58 dB at a population size of 100. The difference is 2.19 dB, as a result. Also, Table 2 shows the PAPR for the AGA SLM OFDM system, which began at 7.86 dB at a population size of 10 and ended at 6.86 dB at a population size of 100. Therefore, there is a 1.01 dB difference. The AGA SLM OFDM system reduced the time from 0.95 s at a population size of 10 to 6.46 s at a population size of 100. Therefore, 5.51 s separate the two times. Therefore, there is a maximum PAPR decrease of 1.18 dB between the GA SLM OFDM system and the proposed AGA SLM OFDM system (2.19 dB − 1.01 dB = 1.18 dB). But in terms of time reduction, the proposed AGA SLM OFDM system and the AGA SLM OFDM system are separated by a maximum of 380.32 s − 15.51 s = 364.81 s. Finally, while the highest time reduction is 364.81 s, which is a significant time saving, the maximum PAPR decrease is just 1.18 dB, which is a tiny PAPR reduction. The suggested AGA SLM OFDM system’s primary contribution is a time savings. Table 2 indicates that the best results of the proposed AGA-SLM-OFDM scheme can be achieved at a population size equal to 100, which satisfied a time reduction of 98.33% compared with the conventional GA-SLM-OFDM scheme.

Table 3 shows a comparison between the AGA-SLM-OFDM and GA-SLM-OFDM at five iterations and different population sizes. As shown in this table, at a population size equal to 10 and five iterations the computation time for the proposed AGA-SLM-OFDM is 2.17 s, and for GA-SLM-OFDM it is 8.98 s. The time reduction of the proposed AGA-SLM-OFDM scheme is equal to 75.84% compared to the GA-SLM-OFDM scheme. Table 3 indicates that the best results of the proposed AGA-SLM-OFDM scheme at five iterations can be achieved at a population size equal to 100, which satisfied a time reduction of 97.61% compared with the conventional GA-SLM-OFDM scheme. In addition, Table 4 shows that the best results of the proposed AGA-SLM-OFDM scheme at 10 iterations can be achieved at population size equal to 100, which satisfied a time reduction of 97.17% compared with the conventional GA-SLM-OFDM scheme.

**Figure 4:**
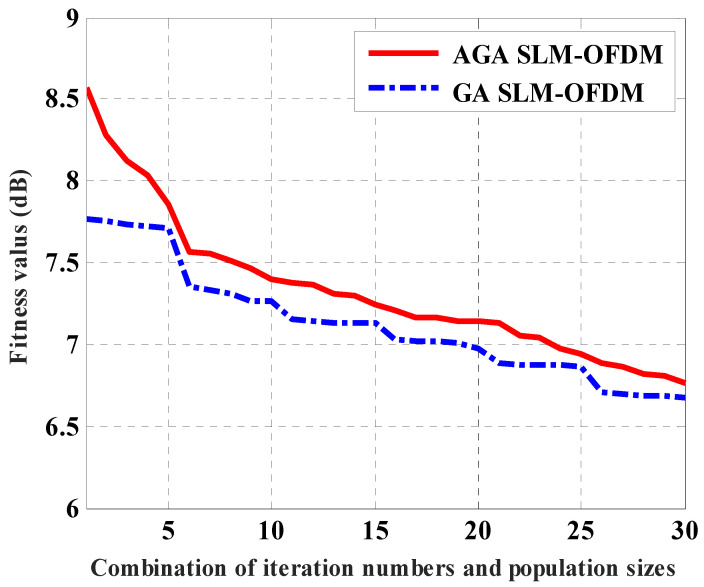
Average fitness values for the proposed AGA SLM-OFDM technique against the conventional GA SLM-OFDM, for the 30 different combinations.

**Figure 5:**
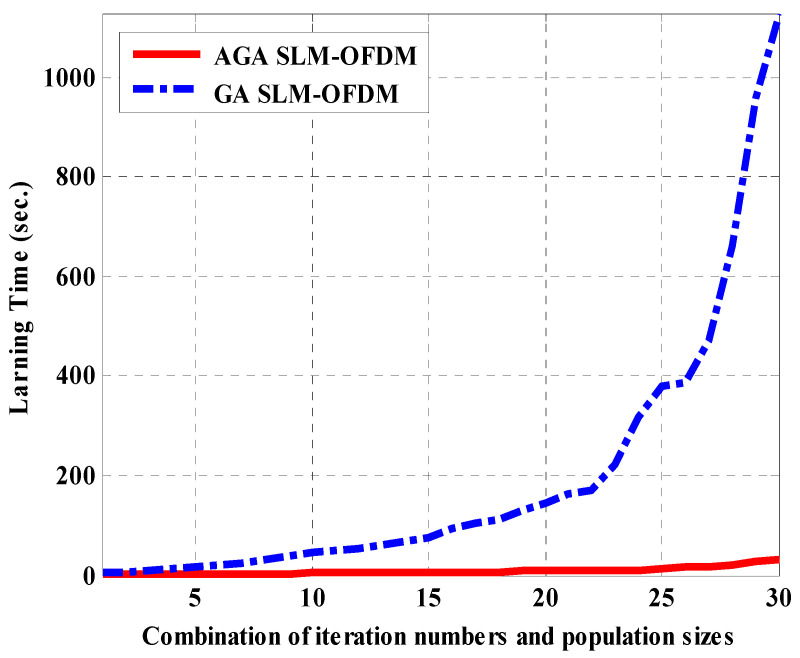
Average learning time values for the proposed AGA SLM-OFDM technique against the conventional GA SLM-OFDM, for the 30 different combinations.

Figure 4 shows the average fitness values of the proposed AGA SLM-OFDM technique against the conventional GA SLM-OFDM, for the 30 different combinations. From Figure 4, we can see that there is little difference in the PAPR (about 0.12 dB) between the two techniques. Figure 5 shows the average learning time values of the proposed AGA SLM-OFDM technique against the conventional GA SLM-OFDM, for the 30 different combinations. From Figure 5, we can see that as the population size and the number of iterations increases, the learning time of the conventional GA increases rapidly with respect to the proposed AGA. This result shows us the advantage of using the on-line tuning of AGA over the off-line tuning of conventional GA [20], as discussed in Section 3.

We find that when the number of iterations is equal to two and the population size is equal to 40, the improvement in the average fitness value is 1.8% (2%); therefore, we choose the number of iterations to be two and the population size to be 40. Note that this criterion for choosing the parameters of a GA is applied to all of the GAs used in this letter. The probability of crossover is chosen to be 0.05; therefore, we reproduce 40 × 0.05 = 2 chromosomes using crossover operation. The probability of mutation is chosen to be 0.05. Therefore, we reproduce 40 × 0.05 = 2 chromosomes randomly with mutation operation to avoid a local minimum of the fitness value.

**Figure 6:**
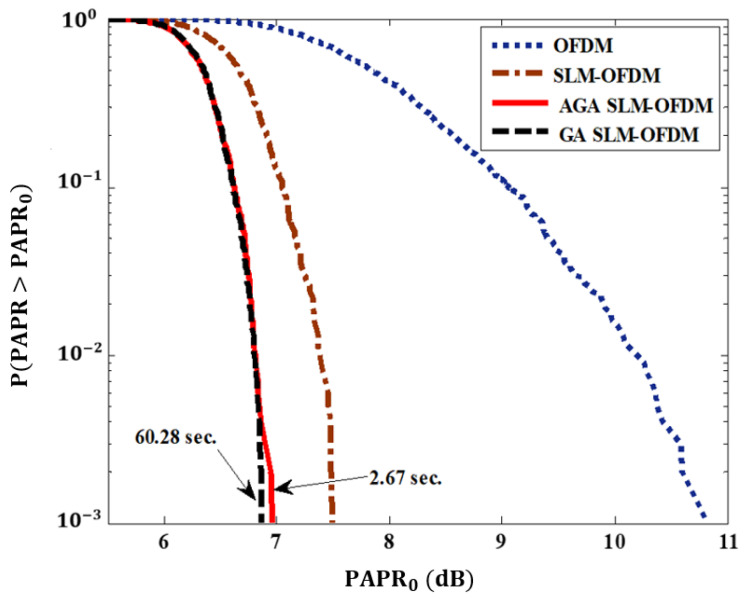
OFDM, SLM-OFDM, GA SLM-OFDM, and the proposed AGA SLM-OFDM PARP_o_ values.

Table 5 shows the learning time and the fitness value for the proposed AGASLM-OFDM compared to the conventional GA SLM-OFDM. From Table 5, we find that the proposed AGA technique reduces the learning time by about 95.56% compared with the learning time of the conventional GA SLM-OFDM with almost the same performance (fitness value). From Figure 6, we find that the proposed AGA SLM-OFDM outperforms the OFDM by about 3.89 dB and the SLM-OFDM by about 0.6 dB, which is slightly smaller than the conventional GA SLM-OFDM by about 0.12 dB.

## 5. Conclusions

This work proposes a new adaptive genetic algorithm based on the SLM-OFDM approach to enhance PAPR and reduce the learning time for identifying the appropriate phase-rotation parameters. In an SLM-OFDM system, the major goal of the AGA is to identify the best phase-rotation factors. The key benefit of the AGA is that it reduces the time required to determine the best phase-rotation factors based on SLM-OFDM. According to the simulation results, learning takes 60.2 s for traditional GA SLM-OFDM and 2.67 s for the suggested AGA SLM-OFDM. The learning time is decreased by around 95.6% using the suggested AGA SLM-OFDM approach. As a result, when compared to the GA SLM-OFDM, the suggested AGA SLM-OFDM exhibits a superb PAPR time reduction. When compared to conventional OFDM, the PAPR of the proposed AGA SLM-OFDM is improved by roughly 3.87 dB. The suggested AGA SLM-OFDM has a PAPR that is around 0.12 dB less than the typical GA SLM-OFDM.

## Figures and Tables

**Table 1:** Simulation parameters.

System Parameters	Value
Number of subcarriers	128
Modulation type	QPSK
Phase rotations	(1, *j*, −1, −*j*)
Sub-block size	16
Population size	40
Number of iterations	2
Mutation probability	0.05
Crossover probability	0.05

**Table 2:** Comparison between the AGA-SLM-OFDM and GA-SLM-OFDM at two iterations and different population sizes.

Population Size	No. of Iter.	2		
	Scheme	AGA	GA	Saving
10	Time (s)	0.95	5.58	46.30%
	PAPR (dB)	7.86	7.77	
20	Time (s)	1.61	18.7	91.40%
	PAPR (dB)	7.51	7.33	
30	Time (s)	2.29	39.1	94.14%
	PAPR (dB)	7.24	7.13	
40	Time (s)	2.67	60.28	95.56%
	PAPR (dB)	6.93	6.81	
60	Time (s)	3.78	131.7	96.98%
	PAPR (dB)	7.04	6.89	
100	Time (s)	6.46	385.9	98.33%
	PAPR (dB)	6.86	5.58	

**Table 3:** Comparison between the AGA-SLM-OFDM and GA-SLM-OFDM at five iterations and different population sizes.

Population Size	No. of Iter.	5		
	Scheme	AGA	GA	Saving (%)
10	Time (s)	2.17	8.98	75.84
	PAPR (dB)	8.04	7.72	
20	Time (s)	3.82	31.4	87.83
	PAPR (dB)	7.56	7.41	
30	Time (s)	5.49	66.9	91.79
	PAPR (dB)	7.30	7.14	
40	Time (s)	6.53	103.3	93.68
	PAPR (dB)	7.17	7.03	
60	Time (s)	9.13	223.5	95.91
	PAPR (dB)	7.05	6.88	
100	Time (s)	15.81	662.3	97.61
	PAPR (dB)	6.81	8.98	

**Table 4:** Comparison between the AGA-SLM-OFDM and GA-SLM-OFDM at 10 iterations and different population sizes.

Population Size	No. of Iter.	10		
	Scheme	AGA	GA	Saving (%)
10	Time (s)	4.35	15.51	71.95
	PAPR (dB)	8.28	7.71	
20	Time (s)	7.52	52.86	85.77
	PAPR (dB)	7.47	7.31	
30	Time (s)	10.74	110.9	90.31
	PAPR (dB)	7.37	7.15	
40	Time (s)	12.13	171.6	92.93
	PAPR (dB)	7.14	7.01	
60	Time (s)	18.61	378.4	95.08
	PAPR (dB)	6.94	6.86	
100	Time (s)	31.93	1128	97.17
	PAPR (dB)	6.82	6.51	

**Table 5:** Learning times and fitness values for the proposed AGA SLM-OFDM and conventional GA SLM-OFDM.

System	Learning Time (s)	Fitness Value (dB)
Proposed AGA SLM-OFDM	2.67	6.93
Conventional GA SLM-OFDM	60.28	6.81

## Data Availability

Data are contained within the article.
